# Automatic Vehicle Fueling System using PLC Controlled Robotic Arm

**DOI:** 10.12688/f1000research.73674.1

**Published:** 2022-09-06

**Authors:** Chitra Venugopal, Bhuvaneswari Thangavel

**Affiliations:** 1 Electrical Engineering and Renewable Energy, Oregon Institute of Technology, Wilsonville, OREGON, 97070, USA; 2 Faculty of Engineering, Multimedia University, Melaka, Malaysia

**Keywords:** PLC, LogixPro, Automatic Vehicle Fueling System, Ladder Logic, Pressure sensor, fuel pump, fuel cap, FASS

## Abstract

The objective of this research is to design and develop a robotic arm for an automatic fuelling system using a PLC LogixPro simulation. The system includes the “FASS” concept, which is Fast, Accurate, Safe and Simple, to allow car users to have an efficient fuel filling system.

The design concept consists of three processes – identification of the vehicle, payment, and filling with the fuel. The first process identifies the presence of the car by the in-floor weight sensors. The weight sensor identifies the car, locks it in position, and activates the payment system. The second process activates the payment system. After payment is completed, the fuel cap will be opened to enable the system to start filling the fuel. If the payment doesn’t go through the car will be released, manual operation will be initialized, and the entire system will be reset. A timer is included in the payment section to process the payment. In the third process, the filling arm is extended to the car, the fuel cap is opened, the fuel pump is inserted into the tank, and fuel is directed into the tank. Once the tank is full, filling is stopped, the pump is ejected, the fuel cap is closed, and the arm returned back to its position. Thus, an automatic vehicle fuelling system is created to overcome the problems of poor safety and longer waiting time during peak hours.

The fuel cap is activated and deactivated by pressure and the sensor filler is stopped by a level sensor. The pump insert is activated and deactivated by a photosensor.

## 1. Introduction

The COVID-19 pandemic has put a spotlight on the role of automation in businesses. Since human contact spreads the disease, machines now have a protective role. This has accelerated the number of robots in industrial applications. The number of vehicles on roads is increasing every day. As fuel stations are operated manually delays are caused and there are long queues, especially in front of gas pumps. During fuelling, drivers must expose themselves to extreme hot or cold temperatures and may also come into contact with dangerous fumes. Thus, an automatic vehicle fuelling system is proposed to overcome problems like poor safety, longer waiting times during peak hours, and the spread of COVID-19.

Petroleum products are expensive. Hence the proper use and distribution of petroleum products is an important task in industries. Fuel stations are built to dispense gasoline and diesel into vehicles for commercial and non-commercial purposes.

In a study by Edward ^1^ to automate the fuel pumping system, an RFID based automated petrol pump system was proposed. In this, the RFID system is used to implement the design tasks sequentially. In Ref. 2 an inventory management procedure is proposed by which the supervisor can find the detailed information of the entity. They can delete the record if they want. Several modules (product cost, staff management) and report forms, like daily pump reports, shift delivery reports, cumulative daily reports, and salary reports are incorporated into the proposed system. It has a password facility for each module for safety purposes.

Fuel stations are operated manually in Bangladesh, leading to delays. Ali Newaz Bahar et al. proposed an automated fuel management system that can maintain the account of the fuel stations to reduce corruption in transactions. ^3^ It prints a receipt automatically after every transaction and can monitor the transactions from a remote place via the internet. ^4^ presents an RFID technology-based fuel dispensing system. The proposed system prevents unauthorized fueling in Baghdad city. Based on the type of vehicle, each registered vehicle will have a specific amount of fuel.

In Ref. 5, a Raspberry Pi based automated fuel pumping system is analyzed. The proposed system aims to confirm the resetting of the transaction every time the fuel pump nozzle is placed back on the dispenser station. In this design, a Raspberry Pi camera and Bluetooth communication are used to reset the dispenser unit when the vehicle passes the dispenser unit. The results show the successful implementation of the automation process.

M.Z.A Rashid et al. proposed an Automatic vehicle fuelling system that utilizes a positioning robot arm. ^6^ They explored a new fuel dispensing system that allowed the robotic arm to move using its search head and extendable nozzle toward the fuel spot of the car sensors are used to identify the location and fuelling position of the vehicle.

In Ref. 7 Wavekar Asrar A et al. aims to design an electronic payment system by creating a prepaid card for petrol stations. In this design, RFID technology is used to automatically pay for petrol dispensed. In Ref. 8 a fuel filling automation process using RFID and GSM technology is discussed. This design uses an RFID card and card reader. When the user swipes the RFID card in the card reader installed in the fuel station, it reads the card information, such as the quantity of fuel to be dispensed, and calculates the amount to deduct from the card. Upon receiving confirmation from the user and the amount remaining in the card, the amount is deducted, and the fuel is dispensed. The GSM technology is used to facilitate the online recharge facility.

Aishwarya Jadhav et al. design a prepaid card for petrol bunk systems and also petrol dispensing systems using RFID technology. ^9^ Use of an unmanned power pump requires less time to operate, is effective, and can be installed anywhere.

Since fuel stations are operated manually, they are time-consuming and require more manpower. In Ref. 10 a self-operated petrol pump is proposed. The use of unmanned petrol pump requires less time to operate. It is effective and can be installed anywhere the customer self-going to avail the services the payment is done by electronic clearing system.

M.B. pranto et al. Proposed an RFID based secure fuel monitoring method with low labour cost. The goal of this system was to avoid the dishonesty of pump labourers & car drivers toward car owners. ^11^ This was achieved by developing a mobile application to trace a vehicle's fuel refill amount, refill cost, current and previous balance of the account & time of transaction. The users need to register themselves first to control their accounts.

In Ref. 12 an IoT-based automated petrol pump system for remote areas is proposed to advantage the petrol station owners. With this proposed smart petrol pump there is no need for a physical operator/person for the distribution of petrol. When a user needs to fill with fuel the vehicle first checks for pricing online in that specific portal designed for this petrol station. Then by selecting the nearby stations and pay for petrol amount. Then he proceeds go the station for filling.

## 2. Methods

### 2.1 Design of the system

The functional diagram of the automatic fuelling system is shown in figure. The functional diagram is divided into three major sections – identification of the vehicle, payment confirmation, and fuel filling which are activated by the sensor inputs and controlled using PLC LogixPro programming.

The system is designed with a robotic arm. The armrests are in position and get activated when the car is parked in the designated space in front of the pumping station. The in-floor weight sensor is activated by the presence of the car which sends a signal to the PLC system indicating that the car has arrived at this pumping station. The PLC system activates the robotic arm and the payment system. Once the payment is successful, the robotic arm is extended to the tank position to fill the fuel. A photoelectric sensor (1) is used to track the position of the arm. When the arm is released from its position, the photoelectric sensor output sends a signal to the PLC to activate the pump insert motor. The opening of the fuel cap is identified by the photoelectric sensor (2). An ultrasonic sensor is used to identify the distance reached by the robotic arm to make sure that the arm has reached the tank position. The ultrasonic sensor and two photoelectric sensors are used to activate the pump insert motor. Once the pump is inserted into the tank, fuel filling happens until the tank level is reached. When the fuel filling is stopped, the pump eject motor is activated, to release the pump from the tank, and the fuel tank cap is closed, the arm is returned back to its original position and is ready for the next fuel filling.

The following algorithm explains the program which controls the entire automation operation.

1. The built-in in-floor weight sensor senses the car and activates the payment sensor.
2. Once the payment is completed, the arm is extended for fuel filling, if the payment is not completed, the system will be reset, and manual operation will be initiated.
3. Fuel arm position sensor is used to activate the fuel arm motor
4. Fuel arm position sensor and fuel arm and fuel arm motor activate the fuel cap opener.
5. Fuel cap open sensor is used to activate the fuel pump insert motor
6. A fuel pump insert sensor is used to start the fuel filling system
7. The level sensor stops the filling once the tank reaches the full level
8. The stop full sensor will eject the pump, the fuel cap will close, the arm will return to its position and the car will be released to move.

*Operation and address*

The input components along with their addresses are shown in Table 1. The output components, along with their addresses, are shown in Table 2.

**Table 1.** Input components along with their addresses.

Operation	Type of device	Port address
Stop	Normally Closed Push Button	I:1/0
Start	Normally Open Push Button	I:3/0
Car Sensor	Weight Sensor	I:1/1
Payment Done	Automatic Signal from the Payment system	I:1/2
Manual Input	Normally open Push Button for Manual Payment system	I:1/4
Fuel Arm Position detector	Ultrasonic Sensor to calculate the distance	I:1/3
Fuel Arm position sensor	Photoelectric sensor 1	I:1/6
Fuel cap sensor	Photoelectric sensor 2	I:1/7
Pump insert sensor	Limit switch	I:1/8
Full level sensor	Fluid level switch	I:1/9

**Table 2.** Output components along with their addresses.

Operation	Type of device	Port address
Lock the Car	Push Button Switch	O:2/0
Payment system	Motor	O:2/1
Fuel arm motor	Motor	O:2/2
Release the Car	Push Button Switch	O:2/3
Fuel cap opener	Motor	O:2/4
Fuel cap close	Motor	O:2/8
Pump insert motor	Motor	O:2/5
Pump eject motor	Motor	O:2/7
Fill fuel	Limit switch	O:2/6

## 3. Results and analysis

The PLC simulation program is simulated using LogixPro 500 ( https://www.plclogix.com) I/O simulator switches.

Step 1: Activate the system – Sensing the Car


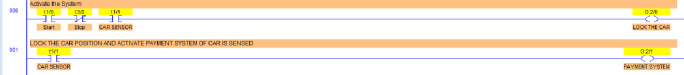


The car sensor is activated by the limit switch. When the car sensor is activated, it activates the payment system.


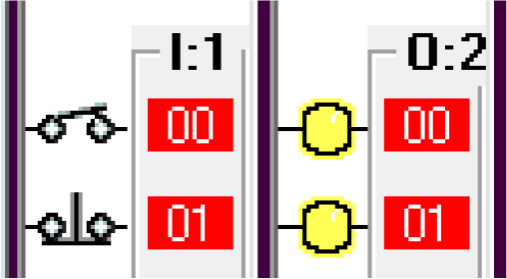


Step 2: The payment system activates the timer.

# 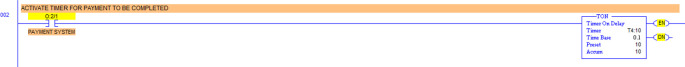


Step 3: When the payment is done at or before the timer bit is done, the fuel arm motor is activated.


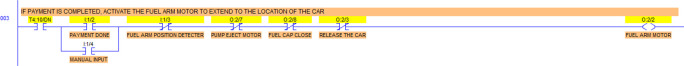


To simulate the system, the ‘payment done’ signal is generated by using a push-button switch in the I/O simulator. To ensure the safety of the system, before activating the fuel arm motor, the fuel arm position detector, pump eject motor, fuel cap close sensor and car release sensor are disabled. Also, manual input is added to bypass the automatic system in case of any error in the automatic system.


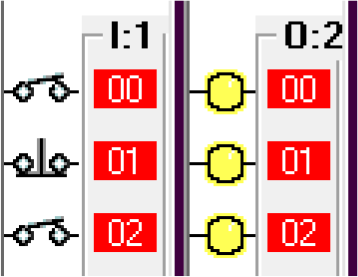


Step 4: If the payment doesn’t go through then release the car and reset the system.


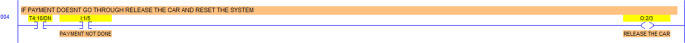


To simulate this condition, the ‘payment not done’ signal is activated using a push-button switch in the I/O simulator.


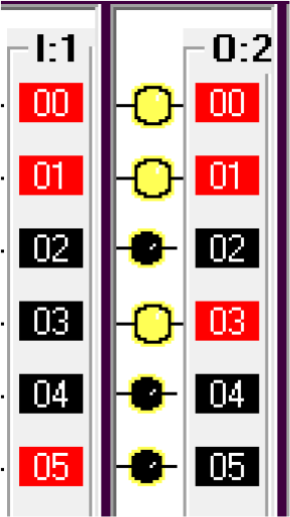


Step 5: Open fuel cap.


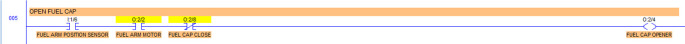


To simulate this condition, the fuel arm position sensor is simulated using a switch in the I/O simulator. For the safety of the system, the fuel cap close condition is used as normally closed in this statement. If the fuel cap is already opened, then the fuel cap opener will not operate.


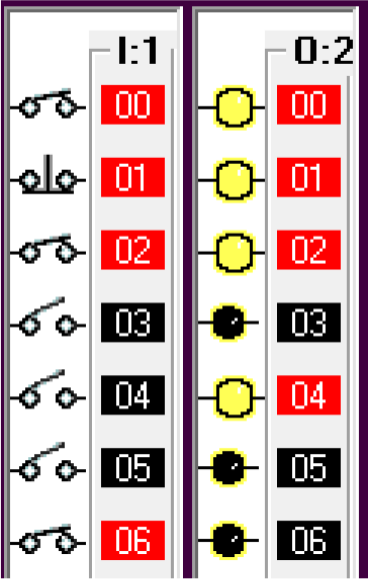


Step 6: Insert Pump.


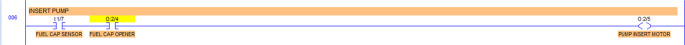


The fuel cap sensor senses the opening of the fuel cap of the car tank. The fuel cap sensor and fuel cap opener activate the fuel arm insert motor. To simulate this step, the fuel cap sensor input is simulated by using a switch in the I/O simulator.


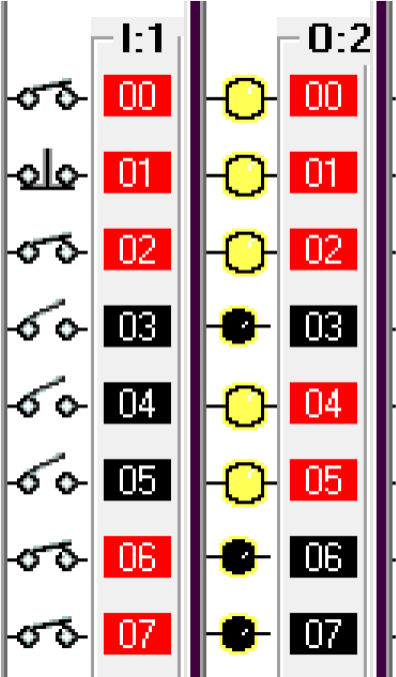


Step 7: Fill Fuel


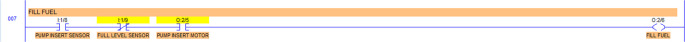


Once the pump is inserted into the tank, it will activate the limit switch which is the pump insert sensor. The full level sensor is a liquid level sensor used to identify the fuel level of the tank. It is used as a normally closed switch as a safety condition to avoid overfill. The pump insert sensor, full level sensor and pump insert motor condition activate the fuel fill start operation. To simulate this condition, the pump insert sensor input is used using a I/O simulator switch.


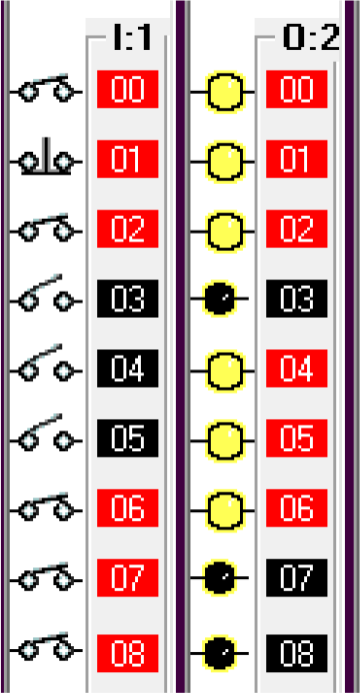


Step 8: Pump Eject

Once the full level is reached, the pump eject motor is activated. The full level sensor is simulated using an I/O simulator switch.


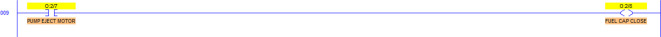



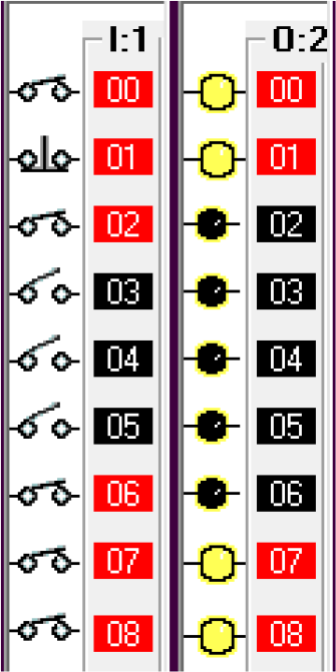


This condition deactivates the pump insert motor, fuel cap opener, fuel arm motor and returns the robotic arm to its original position and releases the car. This can be seen from the deactivated output O:2/2, O:2/3, O:2/4, O:2/4, O:2/5, O:2/6.


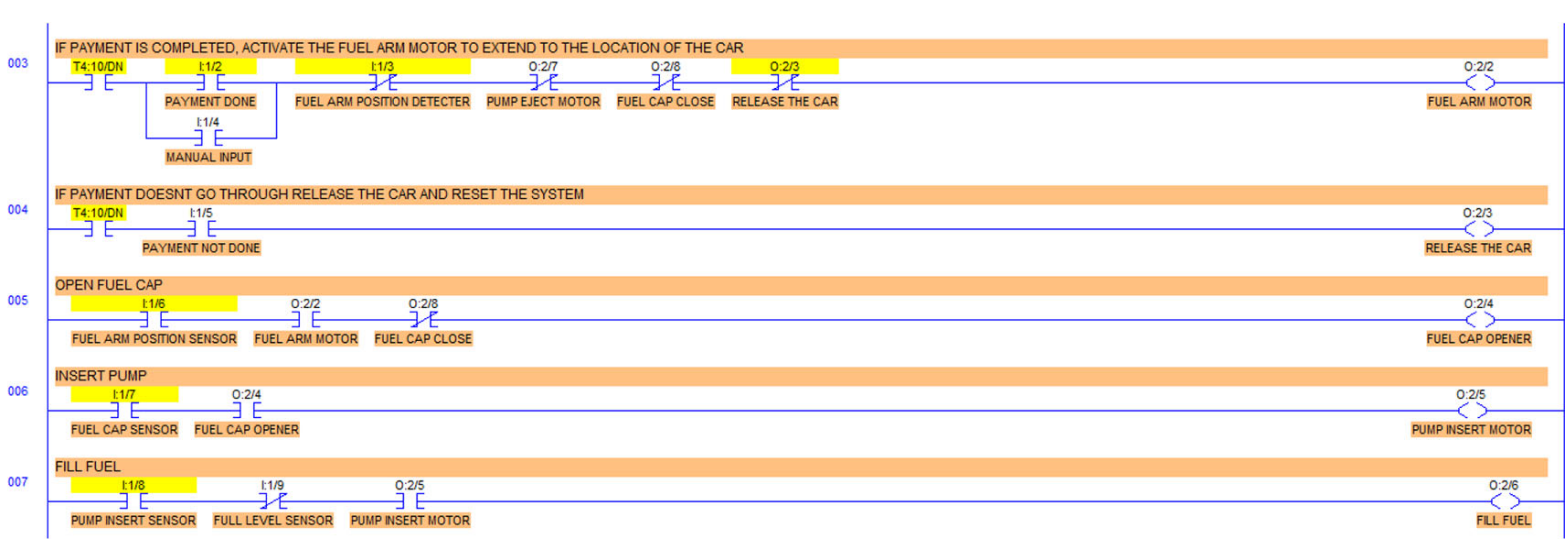


Thus, the algorithm for the automatic fuelling system works in the failsafe mode and achieves the objective successfully.

## 4. Conclusion

The application of PLC in industry is inevitable and is unexplored in the context of fuel filling stations so far. To avoid crimes happening in fuel filling stations due to manual operation and delays caused by manual operation, this automatic fuel filling is suggested. Also, the recent pandemic situation necessitates reducing physical distancing and exchanging of payment systems between customers due to which many fuel stations were closed during pandemic times. Also, the long waiting times during peak hours due to manual operation indicate a dire need for automatic fuel filling systems in this field of engineering. The design of the system considered for this study is explained in this paper. The programming was developed using LogixPro simulation and tested using the I/O simulator. The step-by-step test results were explained. The study shows that the algorithm was implemented successfully and is ready for hardware implementation.

## Data availability statement

All data underlying the results are available as part of the article and no additional source data are required.
