# Supplementary material for: Automatic Vehicle Fueling System using PLC Controlled Robotic Arm - A Simulation Design
Source: F1000Res. 2024 Apr 30;11:1006. Originally published 2022 Sep 6. [Version 2] doi: 10.12688/f1000research.73674.2 (PMC11109568; doi:10.12688/f1000research.73674.2)
Supplement: Peer review history

# Reviewer report: Reviewer response for version 2

**DOI:** 10.5256/f1000research.164528.r270334

**Published:** 2024-05-07

**Authors:** Francisco Javier Folgado

**Affiliations:** 1 University of Extremadura, Extremadura, Spain

The changes made as a result of the previous revision have significantly improved the quality and presentation of the content of the manuscript.

Is the work clearly and accurately presented and does it cite the current literature?

No

If applicable, is the statistical analysis and its interpretation appropriate?

Not applicable

Are all the source data underlying the results available to ensure full reproducibility?

No source data required

Is the study design appropriate and is the work technically sound?

Partly

Are the conclusions drawn adequately supported by the results?

Partly

Are sufficient details of methods and analysis provided to allow replication by others?

Partly

Reviewer Expertise:

Microgrids, Hydrogen, PEM Electrolyzers, Photovoltaic, Renewable Energies, Automation, Internet of Things, Industrial IoT.

I confirm that I have read this submission and believe that I have an appropriate level of expertise to confirm that it is of an acceptable scientific standard.

# Reviewer report: Reviewer response for version 1

**DOI:** 10.5256/f1000research.77340.r166988

**Published:** 2023-04-18

**Authors:** Md. Tabil Ahammed

**Affiliations:** 1 Department of ECE, Khulna University of Engineering and Technology, Khulna, Bangladesh

Great work on designing and developing a robotic arm for an automatic fueling system using a PLC LogixPro simulation. Your use of the FASS concept to ensure that the system is Fast, Accurate, Safe and Simple is commendable. The three-step process you have identified for identification, payment, and fuel filling is well thought out and ensures a smooth user experience. The inclusion of safety features such as the weight sensor, pressure-activated fuel cap, level sensor for filler, and photosensor for pump insert are impressive. This automatic vehicle fueling system is an innovative solution to overcome the problems of poor safety and longer waiting times during peak hours. Overall, this is a well-written report.

Is the work clearly and accurately presented and does it cite the current literature?

Yes

If applicable, is the statistical analysis and its interpretation appropriate?

Yes

Are all the source data underlying the results available to ensure full reproducibility?

Yes

Is the study design appropriate and is the work technically sound?

Yes

Are the conclusions drawn adequately supported by the results?

Yes

Are sufficient details of methods and analysis provided to allow replication by others?

Partly

Reviewer Expertise:

Photonics, Optoelectronics, WSN, Renewable Energy, ML

I confirm that I have read this submission and believe that I have an appropriate level of expertise to confirm that it is of an acceptable scientific standard.

# Reviewer report: Reviewer response for version 1

**DOI:** 10.5256/f1000research.77340.r166991

**Published:** 2023-04-11

**Authors:** S. V. Viraktamath

**Affiliations:** 1 Dept. of Electronics and Communication Engineering, SDM College of Engineering and Technology, Dharwad, India

The paper requires quite a few modifications and the manuscript should be in order to the required format to be accepted. Some recommendations are listed below:

- The contents in the Abstract need to be as per the given format specified. Abbreviations needs to be specified along with their full forms.
- The acronyms must be defined initially in the Section 1. The Literature survey carried out in the same section has various articles along with various approaches carried out doesn’t give a comparative approach thus the survey does not give a summary.
- In the section 2, since there is just one subsection, no need to make a separate section 2.1. Objective of the work carried out in the manuscript is missing.
- The figures in the manuscript does not have any figure numbers along with their description. Also, the figure explanation along with their numbers should be carried out in the content that is followed.

Is the work clearly and accurately presented and does it cite the current literature?

Yes

If applicable, is the statistical analysis and its interpretation appropriate?

Partly

Are all the source data underlying the results available to ensure full reproducibility?

Partly

Is the study design appropriate and is the work technically sound?

Partly

Are the conclusions drawn adequately supported by the results?

Yes

Are sufficient details of methods and analysis provided to allow replication by others?

Partly

Reviewer Expertise:

Error control coding and Image processing

I confirm that I have read this submission and believe that I have an appropriate level of expertise to confirm that it is of an acceptable scientific standard, however I have significant reservations, as outlined above.

# Reviewer report: Reviewer response for version 1

**DOI:** 10.5256/f1000research.77340.r162216

**Published:** 2023-02-13

**Authors:** Francisco Javier Folgado

**Affiliations:** 1 University of Extremadura, Extremadura, Spain

The manuscript covers an interesting R&D topic and fits the scope of the Journal. Nonetheless, the paper requires substantial modifications to its content and format to improve its quality and presentation in order to be accepted. Some considerations and recommendations are listed below.

 First of all, both the title of the paper and the content of the abstract do not accurately reflect the scope of the manuscript. In both, it is implied that all issues related to the development and design of the robotic arm will be addressed. However, the content of the manuscript only focuses on the development of the PLC control algorithm that will command the robot and its testing by means of a simulation. Therefore, the title of the document should be modified to clearly reflect the content of the paper.

 In section 1. Introduction, the acronyms RFID and GSM must be defined before their use. Additionally, references used in the paper are cited in two different formats, a single format should be used for all references.

 Continuing with the Introduction section, this section reviews various articles related to automatic refueling systems. They highlight key aspects of each one or the technologies used. Following this literature review, the reviewer recommends including a paragraph expressly describing the novel aspects of the research carried out. In addition, if appropriate, authors are encouraged to include a comparative table grouping the common aspects of the reviewed works, as well as this paper. In this way, all the aspects of the literature review as well as the novelty of the article are summarized and presented in a visual and simplified way.

 A paragraph should be included detailing the objective and scope of the paper, clearly specifying that only the development of the control algorithm of the automatic refueling system by means of a PLC will be addressed, the results of which will be simulated.

 A common practice in scientific papers consists on providing a brief overview of the structure of the paper at the end of the Introduction. This reviewer suggests including such information for a better presentation.

 In section 2. Methods, the operation of the presented algorithm is described. The heading of subsection 2.1 "Design of the system" should be deleted, since there is no other subsection within section 2.

 The content of this section repeatedly describes how the algorithm works. Firstly, by means of the second paragraph of the section. Secondly, by means of a step-by-step description of the algorithm. In order to eliminate this repetition and to enhance the explanation of the algorithm, the reviewer recommends eliminating the step-by-step description and replacing it with a flowchart-like figure that shows each of these steps in a connected and orderly manner.

 The sentence " Operation and address" should be deleted.

 It is recommended to relocate the phrase " The input components along with their addresses are shown in Table 1. The output components, along with their addresses, are shown in Table 2." before displaying the tables.

 Section 3. Results and analysis presents the simulation process of the developed algorithm. On the other hand, all the figures shown in this section lack a title or legend. It is required to include an individual title for each of the figures presented in the paper.

 Regarding section 4. Conclusions, it is highly recommended to include some sentences related to future work derived from the results obtained in this research.

Is the work clearly and accurately presented and does it cite the current literature?

No

If applicable, is the statistical analysis and its interpretation appropriate?

Not applicable

Are all the source data underlying the results available to ensure full reproducibility?

No source data required

Is the study design appropriate and is the work technically sound?

Partly

Are the conclusions drawn adequately supported by the results?

Partly

Are sufficient details of methods and analysis provided to allow replication by others?

Partly

Reviewer Expertise:

Microgrids, Hydrogen, PEM Electrolyzers, Photovoltaic, Renewable Energies, Automation, Internet of Things, Industrial IoT.

I confirm that I have read this submission and believe that I have an appropriate level of expertise to state that I do not consider it to be of an acceptable scientific standard, for reasons outlined above.
